# High-Performance Liquid Chromatography with DAD Detection for the Determination of Cannabinoids in Commercial Veterinary CBD Oil

**DOI:** 10.3390/pharmacy12060181

**Published:** 2024-12-02

**Authors:** Zehra Hajrulai-Musliu, Elizabeta Dimitreska Stojkovikj, Dimitar Gusheski, Dea Musliu, Daniel Velkovski

**Affiliations:** 1 Faculty of Veterinary Medicine-Skopje, University “Ss. Cyril and Methodius”, Lazar Pop-Trajkov 5/7, 1000 Skopje, North Macedonia; edimitrieska@fvm.ukim.edu.mk (E.D.S.); vet.velkovski@gmail.com (D.V.); 2 FARMAHEM Dooel, Shar Planina 20, 1060 Skopje, North Macedonia; dimitar.ekolab@farmahem.com.mk; 3 Faculty of Pharmacy, Skopje, University “Ss. Cyril and Methodius”, 1000 Skopje, North Macedonia; deamusliu00@hotmail.com

**Keywords:** CBD oil, cannabinoids, THC, HPLC analysis

## Abstract

The study highlights the need for quality control in evaluating medicinal plant products, especially CBD oils, before market release. Due to varying regulatory requirements, product labeling can sometimes be misleading, especially regarding cannabinoid concentrations such as CBD and THC. This research focused on developing a validated high-performance liquid chromatography (HPLC) method for accurately identifying and quantifying key cannabinoids in Commercial Veterinary CBD Oil. The main compounds identified included Cannabidivarin (CBDV), Cannabidiolic Acid (CBD-A), Cannabigerolic Acid (CBG-A), Cannabigerol (CBG), Cannabidiol (CBD), Tetrahydrocannabivarin (THCV), Cannabinol (CBN), ∆^9^-Tetrahydrocannabinol (d9-THC) ∆8-Tetrahydrocannabinol (d8-THC), Cannabicyclol (CBL), Cannabichromene (CBC), and Tetrahydrocannabinolic Acid (THCA), determined in line with the International Conference on Harmonization’s (ICH) guidelines. The method was validated for linearity, accuracy, precision, limit of detection (LOD), and limit of quantitation (LOQ). It was determined to be linear, with a correlation coefficient (R²) > 0.999. The LOD and LOQ values calculated from the calibration curve ranged from 0.05 to 0.13 and 0.50 to 0.61 µg/mL, respectively. The method also exhibited acceptable precision, with relative standard deviation values lower than or equal to 2%. The method’s accuracy was assessed through recovery percentages and fell within an acceptable range of 98–102 if the RSD was 2%. This study’s rigorous methodology and comprehensive findings significantly contribute to cannabinoid analysis. This validated protocol was used to analyze cannabinoids in 14 commercial veterinary CBD oil products from the Republic of North Macedonia. The performance parameters demonstrated that the method is reliable for quantitatively measuring cannabinoids in CBD oil. The analysis showed that the cannabinoid levels in the products were consistent with the manufacturers’ declared specifications, with no significant discrepancies in labeling.

## 1. Introduction

**Figure 1:**
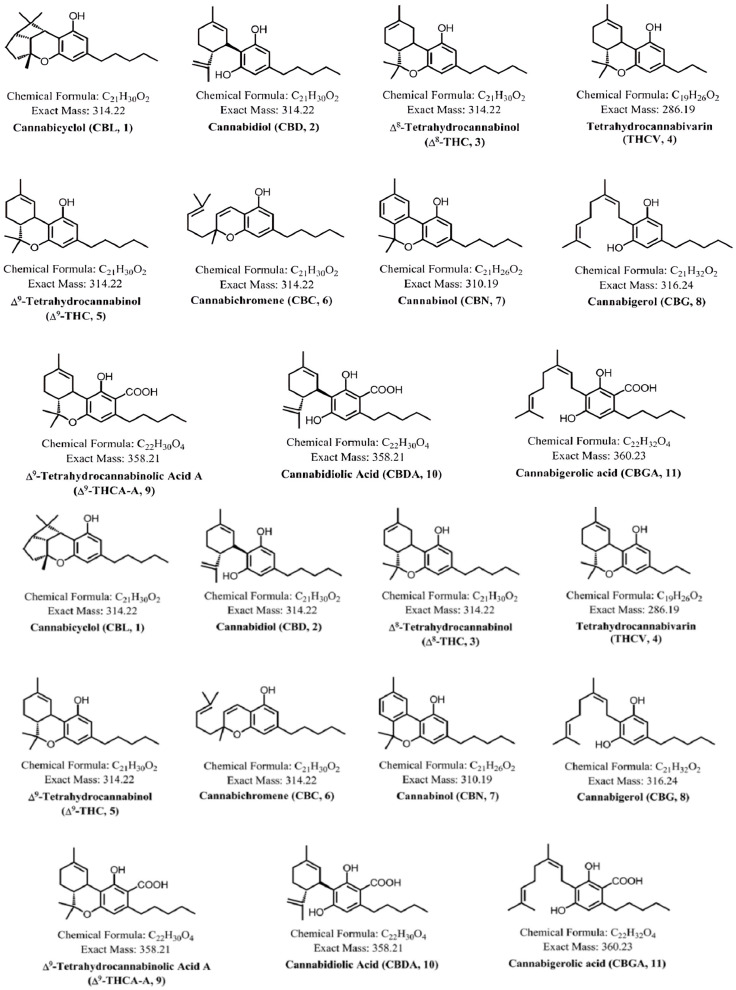
Structures of major cannabinoids in *Cannabis sativa*. Adapted with permission from Reference [15]; published by the *Journal of Forensic Sciences*, 2016.

The use of Cannabis sativa in veterinary medicine has garnered increasing interest in recent years, mainly due to its potential therapeutic effects on various animal conditions. The use of *Cannabis sativa* in veterinary medicine is increasingly recognized for its therapeutic potential in treating a variety of animal conditions. The discovery of the endocannabinoid system in the late 1980s has prompted renewed research into the medicinal properties of cannabis in both human and veterinary medicine [1]. While *Cannabis sativa* is well-known for its psychoactive effects, it contains several cannabinoids, with cannabidiol (CBD) being the most prominent, as shown in Figure 1. CBD appears to be a valuable choice for addressing various animal health challenges. CBD has been noted in managing chronic pain, nausea and vomiting, seizures and convulsions, peripheral neuropathy, and psoriasis; reducing cancer cell growth, inflammatory bowel disorders, bone loss, and hypertension; and lowering blood glucose levels [1,2,3,4,5,6,7,8,9,10,11,12,13,14].

The pharmacological effects of *Cannabis sativa* are not solely attributed to cannabinoids; Terpenes in Cannabis sativa also play a crucial role in its therapeutic effects by interacting with cannabinoid receptors, potentially enhancing the analgesic properties of cannabinoids [16]. Furthermore, the antimicrobial properties of *Cannabis sativa* have been documented, with studies revealing that extracts from the plant exhibit antibacterial activity against various pathogenic strains (“In vitro antibacterial activity of Cannabis sativa leaf extracts to some selective pathogenic bacterial strains,” 2014). This characteristic could be beneficial in treating infections in animals, further expanding the therapeutic scope of cannabis in veterinary medicine [17,18,19]. Despite the growing interest in *Cannabis sativa* for veterinary use, the legal and regulatory landscape remains complex and varies by region. In some areas, cannabis for veterinary purposes is still prohibited, creating challenges for veterinarians and pet owners seeking alternative treatments [20]. As the use of cannabinoids, particularly cannabidiol (CBD), expands, ensuring the safety, efficacy, and consistency of cannabis-derived products is crucial. Variability in cannabinoid concentrations and other compounds can lead to inconsistent therapeutic outcomes and potential adverse reactions [21]. A study by McGrath et al. emphasized the need for standardized formulations to address these issues [22]. Quality control, including third-party testing for contaminants like heavy metals, pesticides, and pathogens, is essential. The American Veterinary Medical Association (AVMA) recommends that only products undergoing comprehensive testing be used in veterinary practice [23,24,25]. Accurate dosing is also critical to avoid under- or overdosing, which can compromise treatment effectiveness. Evidence-based dosing protocols tailored to animals are necessary for consistent results [26,27]. The regulatory framework for cannabis use in animals is still evolving. In many regions, cannabis remains a controlled substance, and the U.S. FDA has not approved any cannabis-derived products for veterinary use [28]. Veterinarians face significant legal challenges regarding the use of cannabis products due to the lack of clear, consistent regulations between state and federal laws, with many states legalizing cannabis for humans but leaving veterinary use in a grey area. Additionally, the absence of FDA-approved cannabis products for animals and concerns about product quality and safety further complicate veterinarians’ ability to legally and ethically prescribe these treatments [29]. In North Macedonia, the use of CBD oil in veterinary medicine remains largely unregulated, with veterinarians facing several concerns related to legal uncertainty, product quality, and ethical responsibility. While medical cannabis has been legalized for human use, veterinary use of CBD is still in its infancy, with no formal approval or clear regulations [30,31]. Moreover, HPLC is a widely used and highly effective analytical technique for the detection and quantification of cannabinoids, as it enables the separation of both neutral cannabinoids (such as CBD and THC) and their acidic precursors (such as CBD-A and THCA) without the need for complex sample derivatization. This is particularly important for CBD oil analysis, where cannabinoids exist in both forms, and precise quantification of both is essential for product quality control. hile GC can be used for cannabinoid analysis, it requires the derivatization of acidic cannabinoids, which adds complexity to the analysis and may lead to sample degradation [32,33].

This work aims to implement and validate an HPLC method for detecting and quantifying cannabinoids in CBD oil, ensuring accurate and reliable results for routine analysis. The validation of an accurate analytical method for analyzing cannabinoids in commercially available products intended for veterinary use is essential for ensuring product safety and efficacy. High-Performance Liquid Chromatography (HPLC) is a widely accepted technique for this purpose, allowing for the precise quantification of cannabinoids such as cannabidiol (CBD) and tetrahydrocannabinol (THC). This work aims to establish a validated HPLC method and compare the analytical results with the declared cannabinoid concentrations on product labels.

## 2. Materials and Methods

### 2.1. Chemicals and Materials

Distilled water (LC Grade), Methanol (LC Grade), and Acetonitrile (LC Grade) were purchased from Merck KGaA, Darmstadt, Germany; certified reference material, which contains 10 components of phytocannabinoid (Δ^9^-Tetrahydrocannabinol (Δ^9^-THC), Δ^8^-Tetrahydrocannabinol (Δ^8^-THC), Tetrahydrocannabinolic Acid (THCA), Cannabinol (CBN), Cannabidiol (CBD), Cannabidioliolic Acid (CBD-A), (±)-Cannabichromene (CBC), Cannabidivarin (CBDV), Cannabigerol (CBG) and Cannabigerolic acid (CBG-A), Mixture 10 (CRM) Cayman A, solution in acetonitrile 250 µg/mL of each compound, certified reference material, which contains (±)-Cannabicyclol (CBL) with concentration of 1 mg/mL ± 0.01 mg/mL in acetonitrile, and certified reference material, which contains Tetrahydrocannabivarin (THCV) with a concentration of 1 mg/mL ± 0.09 mg/mL in acetonitrile were purchased from Chemical Co. 1180 E. Ellsworth Rd. Ann Arbor, MI 48108, USA.

### 2.2. HPLC-Analysis

**Figure 2:**
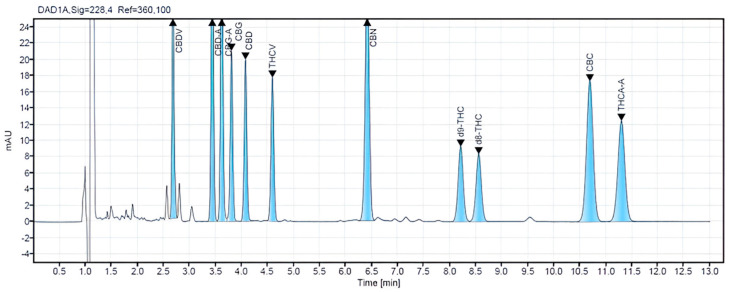
HPLC-DAD chromatogram of CRM 11 mixture standards with a concentration of 25 µg/mL.

The analysis was performed using the Agilent’s 1260 Infinity II LC System, Agilent technologies, Waldbronn, Germany, equipped with a Diode Array Detector (DAD) featuring a wavelength at 228 nm and a Binary Pump (BP) that facilitates the flow of the mobile phase through the instrument at a maximum operating pressure of 600 bar. Chromatographic separation was performed using an Agilent column (Poroshell 120 EC-18, 150 × 3.0 mm, 2.7 µm). The column temperature is 30 °C, and the autosampler temperature is ambient. The separation is isocratic, with a flow rate of 0.6 mL/min, with two mobile phases: 25:75; water with 0.1% formic acid; acetonitrile with 0.1% formic acid.The injection volume is ten μL. This ensured the elution of all cannabinoids with baseline separation in as little as twelve minutes. The last cannabinoid, THCA, was eluted at approximately 11.3 min (see Figure 2).

### 2.3. Sample Preparation 

Using a volumetric automatic pipette for easier sample handling, approximately 100 μL of homogenized cannabis oil was transferred into a 10 mL volumetric flask. The mass of the sample was then measured and recorded for use in calculating the weight percentage of each cannabinoid. The flask was filled halfway with methanol, sealed, and placed in an ultrasonic bath set to 30 °C for 5 min with the sweep function enabled. After the bath, the solution was checked to dissolve the oil completely. The flask was then filled to the 10 mL mark with additional methanol. The solution was stirred and filtered into a glass flask through a 0.2 μm RC filter. Finally, 100 μL of the filtrate was transferred to a vial and diluted with 900 μL of methanol. The vial was vortexed, capped, stirred, and placed on the vial rack of an Agilent system 1260 Infinity II LC, Agilent Technologies, Waldbronn, Germany... The final sample in the vial had a dilution factor of approximately 1000, although this may vary depending on the sample mass and dilution steps. 

### 2.4. Calibration Curve Preparation 

We prepared eight calibration standards at varying concentrations to generate the calibration curve. These standards were derived from an initial 10 mixed CRM solution containing multiple cannabinoids at approximately 100 µg/mL. For cannabicyclol (CBL) and tetrahydrocannabivarin (THCV), separate calibration standards were prepared using their CRMs, each at approximately 100 µg/mL. The standards for the 10 mixed CRM covered a concentration range from approximately 0.1 µg/mL to 85 µg/mL. Likewise, for CBL and THCV, calibration standards were prepared within the same concentration range of 0.1 µg/mL to 85 µg/mL. After preparing the standards, we need to calculate the exact concentration of each cannabinoid in the solutions, considering the concentration and purity declared in the quality certificate of the CRM used. For example, let us take 400 μL from a CBD CRM with a declared concentration of 250 ug/mL with a corrected purity of 99.42% and dissolve to 1 mL with methanol, the final solution. The calculated concentrations for each cannabinoid are then inserted into the calibration table for each level. A crucial requirement for constructing a precise and accurate calibration curve is that the correlation factor must be greater than or equal to 0.999 (R^2^ ≥ 0.999) for each cannabinoid. We verify the calibration curve through the System Suitability Standard, which must have a concentration of approximately 25 µg/mL as read from the calibration curve. If a significantly higher or lower value is obtained, then it is mandatory to construct a new calibration curve with newly prepared standards.

## 3. Results

The analytical method for determining cannabinoid content, described in the German Pharmacopoeia, was applied to analyze various cannabis products and validated according to ICH guidelines [32,33]. Based on HPLC with DAD detection, the method was specifically developed to determine CBD content in the hemp oil and ensure its compliance with declared values. Key validation parameters included system suitability, linearity, accuracy, precision, detection limit (LOD), and quantification limit (LOQ). The validation results are summarized in Table 1, Table 2 and Table 3, demonstrating the robustness and reliability of the method.

### 3.1. Method Validation

#### 3.1.1. Linearity

The linearity of a method is its ability to produce test results that are directly proportional to the concentration or amount of sample within a given range. The relationship between the detector response (peak area) and the sample concentration is used for HPLC methods to determine linearity. The linear representation of this dependence function implies drawing a regression line with the lowest possible residual value for the selected concentration levels. Values on the x-axis (concentration/amount) are considered values with a tiny error justified by the low variability in the preparation of analytical standards about the response of the analytical method represented on the y-axis (area of chromatographic peaks). All standards were prepared by diluting directly from the first standard, Std stock with C ≈ 100 µg/mL. The calibration curve was constructed for each cannabinoid component separately. For this purpose, it is necessary to prepare eight standard solutions containing all 12 cannabinoids, with 0.1–85 µg/mL concentrations. The method was found to be linear for all eight different concentrations. Correlation coefficients (R^2^) for each individual cannabinoid were close to 1, showing good linear correlation (R^2^ > 0.999), as shown in Table 1.

#### 3.1.2. Limit of Detection (LOD) and Limit of Quantification (LOQ)

The limit of detection is the smallest amount in the test sample that can be reliably detected using the appropriate method. The detection limit is determined according to the formula:

*LOD* = 3 *N*/s

The ratio between the noise level (*N*) and the peak height of the cannabinoid is determined from the chromatogram itself, where the peak signal from the cannabinoid should be three times the noise signal (where s is the standard deviation of the regression line and is the slope coefficient of the calibration curve).

The limit of quantification is usually taken to be ten times the signal-to-noise ratio:

*LOQ* = 10 *N*/s

It should also be emphasized that the quoted LOD and LOQ values do not consider the possibility of achieving lower detection limits by the concentration of the obtained extracts. The limit of detection (LOD) and limit of quantification (LOQ) for each cannabinoid was calculated using the mean, standard deviation, and slope from the regression analysis (Table 2). The obtained values are comparable to the results of similar methods described in the literature, confirming the suitability and sensitivity of the technique.

#### 3.1.3. Accuracy

The accuracy of the analytical method is defined as the match between the mean value obtained during the experiment and the accepted reference value. Determining this parameter allows for estimating the effect of the systematic error of the method on the final result. The analytical yield expresses the accuracy when no certified reference material exists. The analytical yield is obtained when enriching negative samples with a standard solution at three concentration levels. It represents the ratio between the obtained and added concentration values, expressed in percent. The accuracy is determined by analyzing the enrichment of oil samples with six repetitions at three different concentrations. The results are shown in Table 3. Analytical yield percentages ranged from 98 to 102% for all cannabinoids (acceptance criteria: 90–110% according to ICH guidelines). Analytical yield data show the high extraction efficiency for cannabinoids from CBD oil, according to the applied method.

#### 3.1.4. Precision

According to ICH, precision is the closeness between the results obtained from a series of measurements made on samples from the same homogeneous source under prescribed conditions. The precision can be expressed as repeatability—where analyses are performed under identical conditions in a short period; intermediate precision—where additional random effects of the working environment are included over a more extended period (days, weeks); and reproducibility—as precision between different laboratories. From the data obtained for six repetitions, mean value, standard deviation, and relative standard deviation are calculated for all analyzed samples. The RSD was 0.82 for (cannabigerol-CBG) while for (cannabidivarin CBDV), 1.59%, with the results shown in Table 2.

#### 3.1.5. Suitability of the HPLC System

In total, 1.0 mL CBD standard solution was diluted with 10 mL acetonitrile in a volumetric flask (10 μg/mL CBD concentration standard mixture) used for the assay method. This solution was used to test the system’s suitability, where six injections were made, which determined the number of theoretical noise floors, peak spreading factor, resolution between peaks, and reproducibility (percent RSD of retention time, area, and height of the peak for six injections). The values obtained for the resolution between the peaks of CBD, Δ^9^-THC, and Δ^8^ -THC in the chromatograms after applying the solution to check the system’s suitability indicate an excellent separation of the components (Table 3). The repeatability of the system is satisfactory (RSD ≤ 1%).

### 3.2. Analysis of Oil Samples

A comprehensive analysis was conducted on 14 different commercial veterinary CBD oils in North Macedonia to evaluate their cannabinoid concentrations and quality control standards. This limited sample size highlights the nascent stage of the veterinary CBD oil market in the region, with the chosen products representing a diverse range of brands, formulations, and price points. Despite the market’s ongoing development, the findings shed light on the quality and regulatory standards of CBD products accessible to consumers locally. The selected samples were carefully sourced from reputable veterinary pharmacies and were stored in their original bottles at a consistent temperature of +4 °C until they underwent detailed analysis. This study provides insight into the current offerings and identifies potential areas for improvement in product quality and regulation. All samples were analyzed using a validated HPLC method to ensure accurate and precise results. The CBD concentration level of the analyzed samples for veterinary oils ranges from 1.4% to 21.20%. In the samples with ordinal numbers 3, 4, 7, 8, 13, and 14, low concentrations of CBG were detected (0.54–0.03%). For CBC in samples 13 and 14, 0.05% was detected, while d^9^-THC was detected in concentrations of 0.04% in samples 13 and 14. Chromatograms of a blank sample and CBD oil extracts containing different concentrations of cannabinoids are presented in Figure 2. Cannabinoid concentrations observed in veterinary oil samples ranged from non-detectable (N/D) to LOQ for CBC, CBG, and d^9^-THC. This may not be too surprising since it is common knowledge that some of these cannabinoids are present at relatively low levels in cannabis and hemp. The obtained results from the analyzed samples in this research are shown in (Table 4).

The cannabinoids listed as ‘no declared values’ were detected in the products but were not declared on the labels. Their presence ranged from absent (‘not detected’) to below the limit of quantification, as reported on the product certificates (Table 4).

## 4. Discussion

FDA-approved drugs provide patients with confidence and safety in the products. Without regulatory scrutiny, many small and large companies are entering the business of manufacturing medical hemp products even though there are no documented clinical benefits from such treatments (U.S. Food and Drug Administration (FDA) [28,31,34]. The “product” is often an extract of an unproven compound that may or may not be beneficial or safe for the patient, especially in the absence of regulatory standards for cannabinoid content, pesticide, or heavy metal contamination [35,36]. On the other hand, CBD regulation in veterinary medicine varies significantly between the EU, the US, and non-EU countries. While CBD is legally available for humans in all these regions, formal approval for its use in animals remains largely absent. In the EU, its use is unregulated and left to individual countries’ discretion. At the same time, in the US, the federal prohibition on cannabis creates a complicated legal landscape despite state-level legalization [37,38]. In non-EU countries like Canada and Australia, the focus is primarily on human-use products, and veterinary use is still restricted. As research advances, future regulatory frameworks may provide clearer guidelines for the use of CBD in veterinary medicine across these regions [39]. 

Our goal in this paper was to analyze a representative set of commercial veterinary CBD oils to determine if the labeled content is accurate. Also, in the absence of any regulatory control, we were interested in knowing whether the THC content of these samples was within legally permissible limits.

The final composition of commercial hemp oil will depend on the type of extraction used and any subsequent purification or treatment of the hemp extract. The relative amounts of these chemicals will also rely on the hemp variety selected [40]. On the other hand, it should be emphasized that cannabinoid receptors are different in humans and animals. In all mammals, these receptors are found in the brain, large organs, and bones. While the human body can produce its endocannabinoids to regulate functions, sometimes their production is disrupted, leading to various diseases. Exogenous cannabinoids, such as CBD oil, can help restore balance. Animals have more receptors in the brain and are, therefore, more sensitive to cannabinoids. They can become toxic more quickly, primarily THC, even at much lower doses. Due to their sensitivity, CBD oils for pets must contain nondetectable THC (AVMA) (2020) [24]. Only products without detectable THC content are registered as veterinary products by the Institute for State Control of Veterinary Biologicals and Medicines in the Czech Republic (SCVBM). Vets using CBD in their practice emphasize the importance of zero THC content [41]. 

On the other hand, there are different analytical techniques for determining and quantifying cannabinoids. Gas chromatography (GC) is the preferred method for cannabinoid analysis. However, chemical derivatization is necessary to prevent the decarboxylation of acidic cannabinoids. Liquid chromatography (LC) has gained popularity with the advent of high-performance liquid chromatography (HPLC) and ultrahigh-pressure liquid chromatography (UHPLC) [42,43]. It enables the determination of cannabinoids in both neutral and acidic forms without the need for derivatization. LC, HPLC, and UHPLC can be paired with various detectors, including fluorescence, mass spectrometry (MS), diode-array detection (DAD), or ultraviolet (UV) detectors. While MS enhances the selectivity and sensitivity of analyses when coupled with HPLC and UHPLC, it comes with higher costs. It requires more specialized expertise to operate, while DAD offers a range of detection. DAD can help to improve specificity because acidic and neutral cannabinoids have different absorption spectrums [44,45,46]. Thus, Andreae et al., 2015, used HPLC-DAD to differentiate between Cannabis sativa chemotypes and extracts of different polarity and profile extracts [47]. 

**Figure 3:**
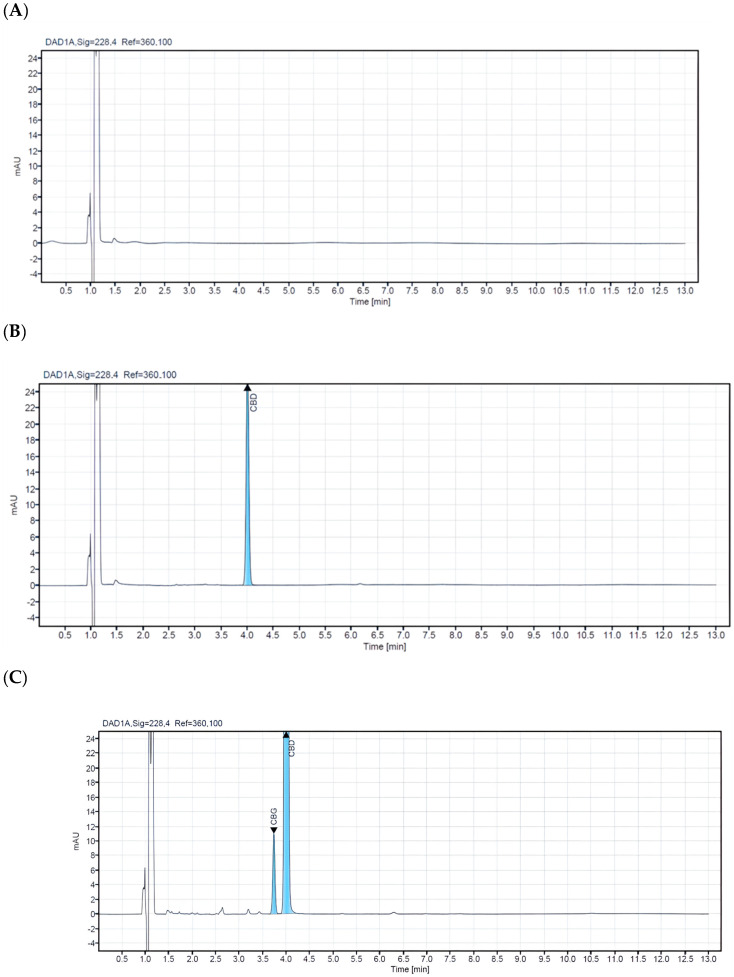

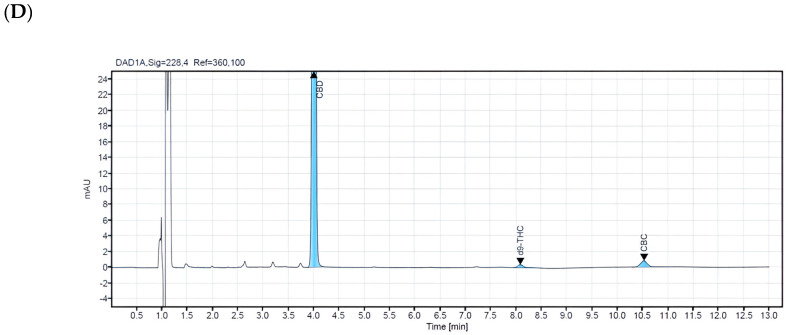
(**A**) HPLC-DAD chromatogram of a negative sample. (**B**) HPLC-DAD chromatogram of an authentic sample of hemp seed oil (oil no. 4). (**C**) HPLC-DAD chromatogram of an authentic sample of hemp seed oil (oil no. 8) and (**D**) HPLC-DAD chromatogram of an authentic sample of hemp seed oil (oil #12).

This paper comprehensively optimizes the HPLC-DAD method, sample preparation, and analysis conditions. We achieved excellent separation of all 12 cannabinoids within 12 min, demonstrating baseline resolution (R > 1.0) between CBD and CBG. Utilizing a carefully selected analytical column and optimized analytical conditions, the isocratic mobile phase consisting of 0.1% aqueous formic acid (A) and 0.1% acetonitrile (B) effectively ensured superior separation of CBD, CBG, and THC, the primary components of hemp oil (see Figure 3). Comprehensive validation confirmed the method’s high precision and accuracy, including specificity, linearity, accuracy, precision, LOD, LOQ, system suitability, and analytical yield. This validated method represents a significant advancement in cannabinoid analysis.

Twelve cannabinoids were analyzed in 14 CBD oil samples, with total CBD concentrations ranging from 16.25 to 206.768 mg/g. CBD was the dominant cannabinoid in all oils, averaging 92.3% of the cannabinoid profile. Declared CBD concentrations ranged from 1.5 to 20%, while measured CBD concentrations ranged from 1.47 to 21.25%. All CBD products prominently declare the concentration of CBD on the packaging, often with higher concentrations offered at higher prices. 

Based on the data presented in this paper, the 14 samples analyzed have CBD concentrations in the range provided by the manufacturers’ certificates (−4.4% < c < 12%). An overview of the determined deviations is shown in Table 4. Regarding the determined content of THC in the products, it was determined that the legal limit of 0.3% was not exceeded in any of them [30]. 

Cannabinoids, mainly when administered at therapeutic doses to animals, exhibit a narrow therapeutic window. This means that even a slight variation in concentration can push the dose beyond the safe range. Certain species may be particularly susceptible to adverse effects such as sedation, hypotension, or gastrointestinal distress if cannabinoid levels exceed the intended dosage, even by a small margin [48]. In our findings, a 4.4% deviation in cannabinoid concentration, while seemingly minor, can have significant clinical consequences, especially in veterinary settings where precise dosing is critical for efficacy and safety. For example, an increase of just 4.4% in cannabinoid concentration could lead to toxicity or unwanted side effects, especially in sensitive species like small mammals or those with pre-existing health conditions. Conversely, a decrease of 4.4% may render the dosage insufficient to achieve the desired therapeutic effect, potentially prolonging symptoms or requiring additional treatments [5,48,49]. Moreover, given that CBD is often marketed for various therapeutic applications in animals, ensuring the accuracy of cannabinoid concentrations is crucial for effective treatment. In summary, our results show that cannabis oil products do not show much variation in terms of cannabinoid content, purity, and labeling.

## Figures and Tables

**Table 1:** Linearity, limit of detection (LOD), and limit of quantification (LOQ) of the method.

Component	Linearity R^2^ ≥ 0.998	LOD at S/N = 3:1	LOQ at S/N = 10:1
Cannabidivarin (CBDV)	0.99947	0.05 μg/mL (S/N = 3.7)	0.50 μg/mL (S/N = 21.2)
Cannabidiolic Acid (CBD-A)	0.99937	0.05 μg/mL (S/N = 7.2)	0.50 μg/mL (S/N = 44.6)
Cannabigerolic Acid (CBG-A)	0.99939	0.05 μg/mL (S/N = 3.9)	0.50 μg/mL (S/N = 19.9)
Cannabigerol (CBG)	0.99945	0.05 μg/mL (S/N = 3.8)	0.50 μg/mL (S/N = 9.6)
Cannabidiol (CBD)	0.99951	0.05 μg/mL (S/N = 4.2)	0.50 μg/mL (S/N = 23.4)
Tetrahydrocannabivarin (THCV)	0.99973	0.05 μg/mL (S/N = 3.3)	0.50 μg/mL (S/N = 20.1)
Cannabinol (CBN)	0.99954	0.05 μg/mL (S/N = 4.8)	0.50 μg/mL (S/N = 29.5)
Δ^9^-Tetrahydrocannabinol (Δ^9^-THC)	0.99951	0.10 μg/mL (S/N = 4.0)	0.50 μg/mL (S/N = 10.1)
Δ^8^-Tetrahydrocannabinol (Δ^8^-THC)	0.99951	0.10 μg/mL (S/N = 3.4)	0.60 μg/mL (S/N = 10.5)
Cannabicyclol (CBL)	0.99959	0.10 μg/mL (S/N = 4.3)	0.61 μg/mL (S/N = 10.5)
Cannabichromene (CBC)	0.99940	0.10 μg/mL (S/N = 3.8)	0.50 μg/mL (S/N = 12.6)
Tetrahydrocannabinolic Acid (THCA-A)	0.99940	0.13 μg/mL (S/N = 3.5)	0.59 μg/mL (S/N = 10.5)

**Table 2:** Accuracy and precision of the analysis method.

Component	Repeatability Within One Day, RSD (%) (n = 18)	Repeatability in 3 Different Days, RSD (%) (n = 3 × 6 = 18)	Reproducibility RSD (%)	Recovery (%)
Cannabidivarin (CBDV)	4.67	4.13	1.59	98.32
Cannabidiolic Acid (CBD-A)	3.76	3.31	1.10	99.75
Cannabigerolic Acid (CBG-A)	3.81	2.97	1.41	98.15
Cannabidiol (CBD)	5.41	4.96	1.49	99.43
Tetrahydrocannabivarin (THCV)	4.43	3.78	1.37	101.24
Cannabigerol (CBG)	4.89	4.61	0.62	98.67
Cannabinol (CBN)	2.59	2.65	0.79	99.37
Δ^9^- Tetrahydrocannabinol (Δ^9^-THC)	3.05	3.17	0.81	98.56
Δ^8^- Tetrahydrocannabinol (Δ^8^-THC)	3.81	3.58	1.07	100.85
Cannabicyclol (CBL)	5.30	4.74	1.01	97.24
Cannabichromene (CBC)	5.12	4.64	1.43	99.51
Tetrahydrocannabinolic Acid (THCA-A)	5.42	5.04	0.84	101.47

**Table 3:** Suitability of the HPLC system.

Component	Rt Precision, RSD * (%)	Area Precision, RSD * (%)	Height Precision, RSD * (%)	Peak Resolution (Rs ≥ 1.5)	Tailing Factor (T ≤ 2.0)	Capacity Factor (k ≥ 2.0)
CBDV	0.11	0.39	0.51	5.343	1.106	3.002
CBD-A	0.11	0.27	0.27	10.074	1.081	1.882
CBG-A	0.12	0.24	0.16	2.141	1.100	2.200
THCV	0.12	0.29	0.25	4.743	1.104	3.760
CBG	0.11	0.24	0.26	2.159	1.042	2.367
CBD	0.11	0.34	0.31	1.511	1.060	2.608
CBN	0.14	0.31	0.39	19.62	1.039	4.678
Δ^9^-THC	0.14	0.23	0.32	11.02	1.048	6.268
Δ^8^-THC	0.13	0.31	0.34	1.852	1.005	6.573
CBL	0.13	0.29	0.32	1.786	1.008	6.342
CBC	0.13	0.50	0.46	9.940	1.030	8.455
THCA-A	0.13	0.72	0.55	2.484	1.030	8.991

* Acceptance criteria ≤ 1%.

**Table 4:** Results from examined values of CBD, CBG, CBC, and d^9^-THC in cannabis oil samples.

Examined Components	CBD (%, *w*/*w*)			CBG (%, *w*/*w*)		CBC (%, *w*/*w*)		Δ^9^-THC (%, *w*/*w*)	
Cannabis Oil Samples	Declared	Examined	Deviation (%)	Declared	Examined	Declared	Examined	Declared	Examined
Sample 1	1.50	1.48	−1.33	n.d. **		n.d. **		n.d. **	
Sample 2	1.50	1.47	−2.00	n.d. **		n.d. **		n.d. **	
Sample 3	1.50	1.46	−2.67	n.d. **	0.04	n.d. **		n.d. **	
Sample 4	1.50	1.46	−2.67	n.d. **	0.03	n.d. **		n.d. **	
Sample 5	5.00	5.07	1.40	n.d. **		n.d. **		n.d. **	
Sample 6	5.00	5.03	0.60	n.d. **		n.d. **		n.d. **	
Sample 7	5.00	4.96	−0.80	n.d. **	0.10	n.d. **		n.d. **	
Sample 8	5.00	4.95	−1.00	n.d. **	0.10	n.d. **		n.d. **	
Sample 9	10.00	9.56	−4.40	n.d. **		n.d. **		n.d. **	
Sample 10	10.00	9.57	−4.30	n.d. **		n.d. **		n.d. **	
Sample 11	10.00	11.20	12.00	n.d. **		n.d. **	0.05	n.d. **	0.04
Sample 12	10.00	11.12	11.20	n.d. **		n.d. **	0.05	n.d. **	0.04
Sample 13	20.00	21.25	6.25	n.d. **	0.54	n.d. **		n.d. **	
Sample 14	20.00	21.19	5.95	n.d. **	0.54	n.d. **		n.d. **	

** n.d.—no declared.

## Data Availability

The original contributions presented in the study are included in the article, further inquiries can be directed to the corresponding author/s. The original contributions are presented in the article.
